# Developing Initial Middle Range Theories in Realist Evaluation: A Case of an Organisational Intervention

**DOI:** 10.3390/ijerph18168360

**Published:** 2021-08-07

**Authors:** Hamid Roodbari, Karina Nielsen, Carolyn Axtell, Susan E. Peters, Glorian Sorensen

**Affiliations:** 1 Institute for Work Psychology, Sheffield University Management School, University of Sheffield, Sheffield S10 2TN, UK; k.m.nielsen@sheffield.ac.uk (K.N.); c.m.axtell@sheffield.ac.uk (C.A.); 2 Department of Social and Behavioral Sciences, Harvard T.H. Chan School of Public Health, Boston, MA 02115, USA; sepeters@hsph.harvard.edu (S.E.P.); glorian_sorensen@dfci.harvard.edu (G.S.); 3 Dana-Farber Cancer Institute, Boston, MA 02215, USA

**Keywords:** realist evaluation, organisational interventions, context–mechanism–outcome configuration, middle range theory, mechanism

## Abstract

(1) Background: Realist evaluation is a promising approach for evaluating organisational interventions. Crucial to realist evaluation is the development and testing of middle range theories (MRTs). MRTs are programme theories that outline how the intervention mechanisms work in a specific context to bring about certain outcomes. To the best of our knowledge, no organisational intervention study has yet developed initial MRTs. This study aimed to develop initial MRTs based on qualitative evidence from the development phase of an organisational intervention in a large multi-national organisation, the US food service industry. (2) Methods: Data were collected through 20 semi-structured interviews with the organisation′s managers, five focus groups with a total of 30 employees, and five worksite observations. Template analysis was used to analyse data. (3) Results: Four initial MRTs were developed based on four mechanisms of participation, leadership commitment, communication, and tailoring the intervention to fit the organisational context to formulate ‘what may work for whom in which circumstances?’ in organisational interventions; (4) Conclusions: Our findings provide insights into ‘how’ and ‘which’ initial MRTs can be developed in organisational interventions.

## 1. Introduction

Organisational interventions are the recommended approach for improving psychosocial working conditions and employees’ health and wellbeing [1,2]. Organisational interventions are ‘planned, behavioural, and theory-based actions that aim to improve employees’ health and wellbeing by changing the way work is designed, organised, and managed’ [3] p. 1030. The organisational intervention literature shows that some organisational interventions have resulted in improvements in psychosocial working conditions and employee health and wellbeing; others have resulted in no effect, and a few even led to a deterioration in psychosocial working conditions and employee health and wellbeing [4,5,6]. Realist evaluation is a promising approach for evaluating organisational interventions [7]. Realist evaluation seeks to answer the important question of ‘what works for whom in which circumstances?’ through an iterative cycle of developing middle range theories (MRTs) and testing these theories [8]. MRTs are programme theories about how the mechanisms of an intervention work in a specific context to bring about certain outcomes [9]. Despite the emerging application of realist evaluation in organisational intervention studies [10,11,12,13], to the best of our knowledge, no organisational intervention study has developed initial MRTs. This study aimed to apply realist evaluation in an organisational intervention study by exploring ‘how’ and ‘which’ initial MRTs can be developed.

**Figure 1:**
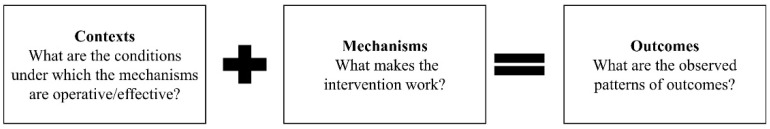
The relationship between contexts, mechanisms, and outcomes in a CMO configuration.

Realist evaluation examines the underlying mechanisms of an intervention (what makes the intervention work?), the contexts under which the mechanisms operate (what are the conditions under which the mechanisms are operative/effective?), and the patterns of outcomes produced (what are the observed patterns of outcomes?) in CMO configurations (Contexts + Mechanisms = Outcomes) [7,9]. Figure 1 depicts the relationship between contexts, mechanisms, and outcomes in a CMO configuration. To evaluate an intervention, realist evaluation involves an iterative cycle that has four steps of (1) developing initial MRTs (i.e., a descriptive format of CMO configurations), (2) planning and implementing the intervention, based on these initial MRTs and collecting empirical data relevant to the mechanisms, contexts, and outcomes of the initial MRTs, (3) analysing and synthesising empirical data based on observed outcomes to formulate empirical MRTs, and (4) testing (i.e., confirming, refuting, or modifying) initial MRTs against the empirical MRTs [8,14].

This study focused on the first step of the realist evaluation cycle; that is, the development of initial MRTs. To develop initial MRTs, realist evaluation requires identifying the most relevant mechanisms in each intervention study [8,15]. In this regard, the organisational intervention literature shows a number of intervention mechanisms that influence the success or failure of the interventions. In a recent review of organisational interventions, Nielsen and Noblet (2018) [16] identified a number of key intervention mechanisms, including tailoring the intervention to fit the organisational context, employees’ participation, and management support. In addition, Schelvis et al. (2016) [17] identified important intervention mechanisms as targeting the right problem to solve in the organisation, employees’ participation, middle management support, senior management support, communication, and employees’ exposure to the intervention activities. Furthermore, Peters et al. (2020) [18] identified four essential intervention process mechanisms as organisation–intervention fit, employees’ participation, communication, and leadership commitment. These authors concluded that if these intervention mechanisms are not triggered/operated, the intended outcomes of interventions may not materialise [16,17,18].

Participation refers to the collective engagement of both employees and their worksite managers in the decision-making process about what and how changes in working conditions can be made in their specific worksite [3]. Leadership commitment refers to the engagement of senior management in the intervention through showing commitment to the intervention and allocating necessary resources to the intervention activities [7]. Communication refers to forming bottom-up and top-down communications between intervention stakeholders about the intervention [19]. Finally, tailoring the intervention to fit the organisational context refers to tailoring intervention components to fit existing policies and procedures, existing working conditions, and individuals in the organisation [20].

Since the success of an organisational intervention depends on the engagement of all organisational members (i.e., employees, line managers, and senior managers), it is important to explore the role of such intervention stakeholders in the change process [21]. Therefore, participation (of both employees and their worksite managers) and leadership commitment are two important process mechanisms that influence the effectiveness of organisational interventions and should be evaluated [16,17]. In addition, since the process and content of communication among the intervention stakeholders influence their awareness of the intervention, engagement in the intervention, and ultimately intervention outcomes, it is important to examine how the intervention stakeholders communicated with each other about the intervention and what kind of information was exchanged among them [21]. Hence, communication about the intervention among the intervention stakeholders is the third key process mechanism that should be evaluated [18]. Fourth, the intervention should fit the culture and working conditions of the intervention stakeholders [21], otherwise, the intervention may be perceived negatively and lead to the failure of the intervention [16]. As such, tailoring the intervention to fit the organisational context is the fourth key process mechanism that should be evaluated [16,18]. In the current study, therefore, we focused on four process mechanisms that have been reported as important mechanisms of organisational interventions: participation, leadership commitment, communication, and tailoring the intervention to fit the organisational context [16,17,18]. We focused on the process mechanisms, rather than contextual factors and outcomes, because the existing literature is less consistent as to what contextual factors and which outcomes are important, only the four mechanisms are clearly stated in the literature. In our study, we allowed exploration of other mechanisms, however, since we defined the above four mechanisms at high levels (e.g., participation mechanism covered both employees and worksite managers), no other mechanisms were identified.

Focusing on the above four mechanisms, we developed four initial MRTs by analysing qualitative data from the development phase of an organisational intervention aiming to improve employees’ health and wellbeing in a large multi-national organisation operating in the US food service industry. We explored how the process mechanisms of participation, leadership commitment, communication, and tailoring the intervention to fit the organisational context may be operated in the intervention study in the food service industry, what contextual factors may influence the operation of such mechanisms, and what outcomes these mechanisms may produce. Based on such findings, we developed CMO configurations and initial MRTs. In this study, therefore, we investigated the following general research questions:

*Research Questions:* How to develop initial MRTs in the organisational intervention? Which initial MRTs can be developed in the organisational intervention?

The contribution of this study is twofold. First, regarding the process of developing initial MRTs (i.e., how to develop initial MRTs in the organisational intervention?), this study addresses the call by Wong et al. (2013, 2016) [22,23] to provide details on how realist evaluation has been used in intervention studies. This article is an example of how to develop initial MRTs as required by realist evaluation. To the best of our knowledge, this article is the first to develop initial MRTs in an organisational intervention [19].

Second, in terms of the contents of initial MRTs (i.e., which initial MRTs can be developed in the organisational intervention?), this study developed four initial MRTs about the key process mechanisms of participation, leadership commitment, communication, and tailoring the intervention to fit the organisational context and their causally related contextual factors and outcomes. These initial MRTs—by analysing organisational stakeholders′ perspectives about how interactions between certain contextual factors and certain intervention mechanisms might produce certain outcomes—improve our understanding of what may work for whom, why, how, and under which circumstances [7]. The novelty of our article, despite previous organisational intervention studies, is linking context–mechanism–outcome elements together in initial MRTs. These initial MRTs create awareness of important intervention mechanisms and their causally related contextual factors and outcomes, and hence provide insights into which data should be collected and analysed when evaluating organisational interventions [7]. This is particularly important in worker populations that have, for example, low levels of autonomy [24], and low wage and immigrant workers [11], such as those employees included in this case study. Researchers and occupational health practitioners can then test (i.e., confirm, refute, or modify) these initial MRTs by using empirical evidence from their interventions [8,9].

## 2. Materials and Methods

### 2.1. Design

The present study was based on formative qualitative research [25] conducted as part of the development phase of the Workplace Organizational Health Study, a proof-of-concept intervention study implemented as a Cluster Randomised Controlled Trial (CRCT) [26]. The formative research was used to identify working conditions to be targeted by the intervention, prioritise intervention outcomes, and identify promising intervention mechanisms [18]. The formative research was conducted during Spring–Summer 2017 in five worksites with between 7 and 30 employees. To avoid contamination [19], the worksites included in the formative research were not included in the later CRCT.

### 2.2. Setting

The study was conducted in a large multi-national organisation operating in the US food service industry. This organisation had worksites that provided food service to corporate clients. The worksites participating in the formative research were located in corporate clients′ premises across Boston, Massachusetts, USA. These worksites were organised by district, based on their geographical location. Each worksite had a specific corporate client (e.g., medical, legal, banking) with specific food service contract terms.

### 2.3. Data Collection

To develop initial MRTs, the research team conducted semi-structured interviews with managers and focus groups with employees and undertook non-participant worksite observations.

#### 2.3.1. Interviews with Managers

Three research team members conducted 20 semi-structured interviews with the organisation′s managers. The organisation generated a list of managers and, on behalf of the research team, sent recruitment letters/emails to its managers to participate in the intervention. At the district level, the research team conducted 11 telephone interviews with 12 district-level managers including district managers and other senior managers (human resources, health and safety, vice presidents, and senior vice presidents). At the worksite level, the research team conducted nine telephone interviews with worksite managers. An open-ended moderator guide was used containing questions about management and leadership perspectives on working conditions, essential elements of the intervention, and possible intervention outcomes. Each interview took approximately 35 min (ranging between 25 and 42 min). The research team audiotaped and transcribed all interviews. At the district level, each participant was given an identifier (DM#1–12) and at the worksite level, each participant was given an identifier (GM#1–4, 6, 8, 9, 12, 13).

#### 2.3.2. Focus Groups with Employees

The research team conducted five focus groups with a total of 30 employees. The research team provided flyers to be distributed and a script to be read in both English and Spanish by the worksite manager in a team meeting inviting employees to participate. All focus groups were conducted on-site and to maintain privacy and confidentiality, no managers attended or observed the focus groups. Focus groups took approximately 60 min (ranging between 56 and 60 min). At one worksite, to accommodate the participants and the inability to release all employees from their duties at one time, the intended focus group was changed into four individual interviews; each took approximately 20 min (ranging between 15 and 25 min). A semi-structured focus group guide was used, including questions related to working conditions that impact employees’ health and wellbeing, important aspects of employees’ health and safety (e.g., pain, injury), essential intervention elements, and ideas and perspectives on how working conditions could be improved. The research team audiotaped and transcribed all focus groups. In focus groups, each participant was identified with their corresponding focus group or interview (FG#1–4, Int#1–4).

#### 2.3.3. Non-Participant Worksite Observations by the Research Team

The research team conducted non-participant worksite observations to observe a normal workday at the five participating focus group worksites to triangulate data from the interviews with managers and focus groups with employees [27]. A research team member conducted the observations in August 2018 over a three-to-four-hour period at each worksite before the focus group. Based on the observations, extensive observation field notes were taken that contained identified specific risks in the physical work environment including the customer-facing, kitchen, and storage areas. The research team, then, summarised and discussed the field notes. The observations were highly useful in improving the contextual understanding of the setting and culture of each worksite.

### 2.4. Data Analysis and Synthesis

We used template analysis to analyse our data. This method stands ‘between content analysis where all codes are predetermined… and grounded theory where there is no a priori definition of codes’ [28] p. 118. When coding data using template analysis, an initial template (a set of priori codes) is defined (based on existing knowledge about the study objects), then this initial template is refined as data are analysed [29]. We used template analysis, instead of a grounded theory approach [30], as the recent organisational intervention research has provided the information necessary to develop an initial template [31]. We developed the initial coding template based on Nielsen and Noblet (2018) [16], Schelvis et al. (2016) [17], and Peters et al. (2020) [18]. Therefore, our initial coding template contained four essential intervention process mechanisms; namely participation, leadership commitment, communication, and tailoring the intervention to fit the organisational context.

To refine (i.e., expand) the initial template, we analysed and synthesised the collected qualitative data. To analyse data, first, an experienced qualitative researcher coded data according to (1) promising intervention mechanisms identified by managers and employees, (2) contextual factors including existing working conditions and policies and practices related to health, wellbeing, and safety, and (3) outcomes of interest. A second qualitative researcher from a different discipline and training cross checked the codes against the original transcripts to enhance trustworthiness (peer debriefing). The observation field notes were used to triangulate and confirm the data observed in the interviews and focus groups. Then, the first, second, and third authors examined the initial template against the emerged codes and expanded the initial template to contain intervention mechanisms, contextual factors, and outcomes identified in the data set. Next, following a process of retroduction [32], the first, second, and third authors synthesised data by identifying how the process mechanisms of participation, leadership commitment, communication, and tailoring the intervention to fit the organisational context could be operated in this intervention, what contextual factors may impair or facilitate the activation of each mechanism, and what outcomes each mechanism may produce. In doing so, the analysed data were synthesised into four contextualised CMO configurations that were, then, translated into four initial MRTs using the statement of *‘**if** there are specific contextual factors, **then** specific mechanisms produce specific outcomes’*. Four final templates were developed, representing four initial MRTs, each template focusing on one initial MRT. During the analysis and synthesis process, the first, second, and third authors met regularly to refine the initial MRTs and their corresponding final templates.

### 2.5. Trustworthiness

To ensure the trustworthiness of our qualitative findings, we followed a set of criteria proposed by Lincoln and Guba (1985) [33]. Their criteria of credibility were met through prolonged engagement in the field and peer debriefing. Their criterion of transferability was met by the description of the study design and organisational context. Their criteria of dependability were met by protecting research participants’ confidentiality and drawing on different stakeholders at multiple organisational levels. Finally, their criteria of confirmability were met using template analysis and retroduction and transparent presentation of the data.

## 3. Results

**Figure 2:**
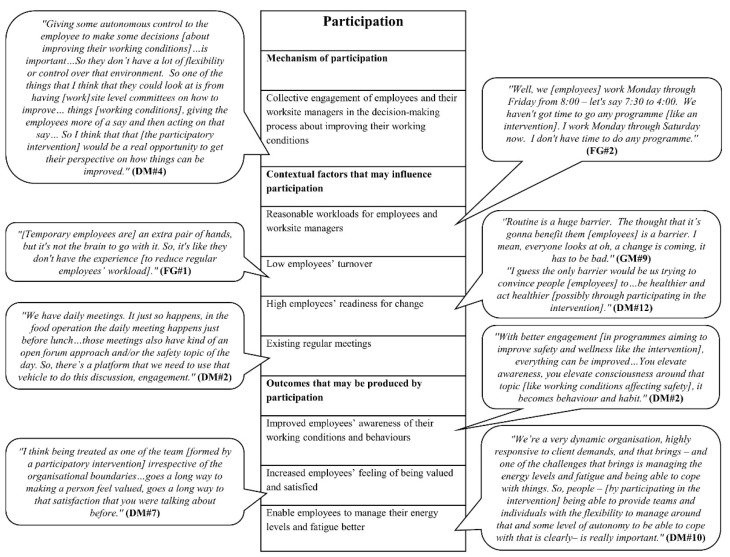
Final template for the initial MRT about participation.

**Figure 3:**
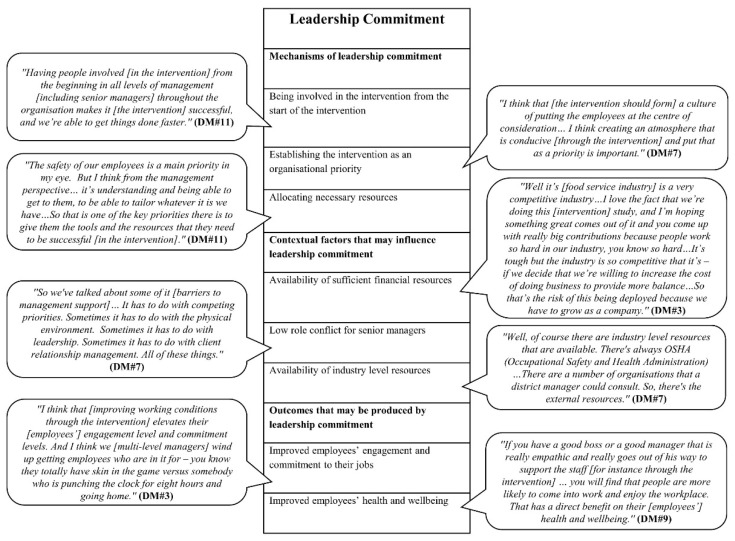
Final template for the initial MRT about leadership commitment.

**Figure 4:**
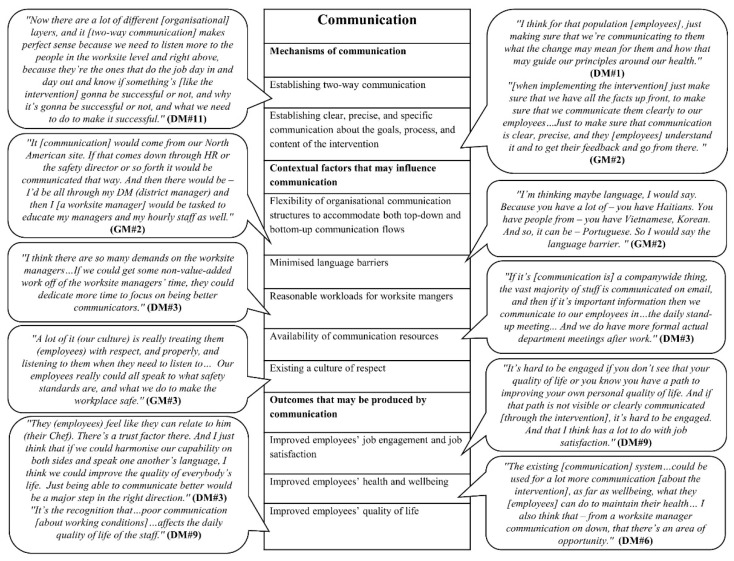
Final template for the initial MRT about communication.

**Figure 5:**
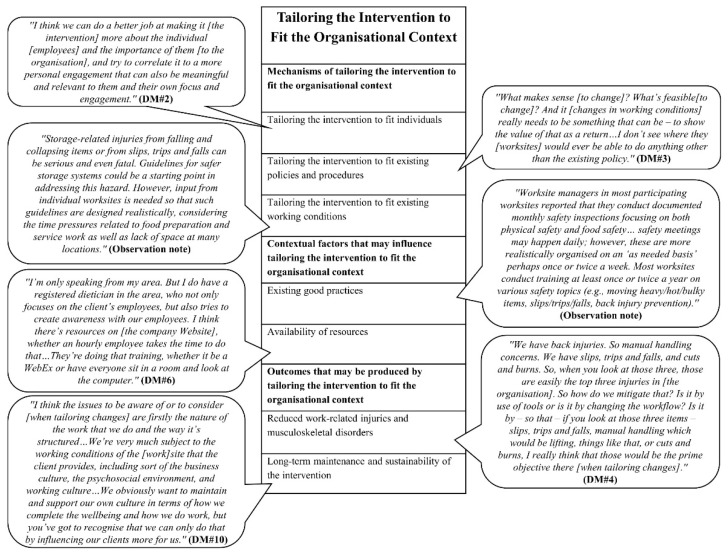
Final template for the initial MRT about tailoring the intervention to fit the organisational context.

We developed four initial MRTs, focusing on four key process mechanisms highlighted in the organisational intervention literature, based on the empirical data from the formative research. In the following, the mechanisms and their causally related contextual factors and outcomes are highlighted; based on them, we developed an initial MRT. The findings are also presented in four figures (Figure 2, Figure 3, Figure 4 and Figure 5). Each figure is a final template of one initial MRT. The text bubbles in the figures represent the relevant quotations/field observation notes from the data.

### 3.1. Initial MRT about Participation

#### 3.1.1. Mechanism of Participation

Our analysed data showed that participation within food service industry intervention may operate through the following key mechanism.

*Mechanism: collective engagement of employees and their worksite managers in the decision-making process concerning improving their working conditions.* Managers at different levels suggested that a mechanism could be introduced, which grants autonomy to employees to, collectively with worksite managers, make decisions about how working conditions could be improved. Employees could be given the opportunity to discuss their physical and psychosocial working conditions with their worksite managers in worksite level committees. As part of this mechanism, employees would offer their perspective on how working conditions could be improved to their worksite managers who could then act on employees’ suggestions.

#### 3.1.2. Contextual Factors That May Influence Participation

Our participants highlighted four contextual factors that may influence participation.

*Contextual factor one: reasonable workloads for employees and worksite managers.* Employees and worksite managers reported high workloads and time pressure as a potential barrier to participation. This means, to trigger participation, employees and worksite managers need reasonable workloads to have the time to participate in the intervention′s activities.

*Contextual factor two: low employees’ turnover.* Worksite managers and employees acknowledged a high level of employees’ turnover in the organisation and reported that the resultant high number of new, temporary employees would have insufficient experience in the food service environment to reduce regular employees’ workload. Therefore, regular employees’ workload would not be decreased to have time to participate in the intervention. This suggests that the level of employee turnover should be so low that regular employees have enough time to participate in the intervention′s activities.

*Contextual factor three: high employee readiness for change.* Managers at different levels highlighted that low employee readiness for change in terms of high routine inertia and lack of motivation would impair participation. This means that, to trigger participation, employee readiness for change should be high.

*Contextual factor four: existing regular meetings*. District-level managers pointed out that existing daily, weekly, biweekly, and monthly meetings in the organisation would provide a platform that would facilitate participation in the intervention.

#### 3.1.3. Outcomes That May Be Produced by Participation

Our participants suggested three outcomes that may be produced by participation.

*Outcome one: improved employee awareness of their working conditions and behaviours.* District-level managers stated that participation in the intervention activities would improve employee awareness of their working conditions and behaviours. In particular, participation would improve employee awareness of their working conditions that would affect their safety and wellness and the working conditions that would make them feel good, safe, comfortable, and confident in their job and in their work environment.

*Outcome two:increased employee feelings of being valued and satisfied.* District-level managers reasoned that participation in the intervention through forming teams and participating in team meetings, in which employees can freely provide their inputs, irrespective of the organisational boundaries, would help employees to feel valued and would improve their satisfaction.

*Outcome three: enable employees to manage their energy levels and fatigue better.* District-level managers pointed out that participation by providing job autonomy to employees regarding task management, decision making on the job, and skills to be able to rotate or work across jobs, as needed, would enable them to manage their energy level and fatigue. They highlighted that fatigue was associated with the schedules of the employees, including the early hours of their shifts, working multiple jobs, and the pace of the workday; hence, allowing employees to schedule their tasks would enable them to match their tasks with their energy level, which would ultimately reduce their fatigue.

The above analyses lead to the following initial MRT.

*Initial MRT about participation: ****if*** there are reasonable workloads for employees and worksite managers, the level of employees’ turnover is low, employee readiness for change is high, and there are structures in place, including existing regular meetings (*contextual factors*), ***then*** giving autonomy to employees to, collectively with their worksite managers, make decisions about improving their working conditions (a *mechanism*) will improve employees’ awareness of their working conditions and behaviours, management of their energy levels and fatigue, and their feeling of being valued and satisfied (*outcomes*).

### 3.2. Initial MRT about Leadership Commitment

#### 3.2.1. Mechanisms of Leadership Commitment

Our data showed that leadership commitment may operate in three ways (i.e., three mechanisms) within the food service industry intervention, as outlined below.

*Mechanism one: being involved in the intervention from the start of the intervention*. District-level managers argued that, if senior management (i.e., district and higher-level managers), are not involved in the change process from the beginning, the lower-level managers and employees may choose some change initiatives that need large (financial) resources, and this would make the change process a longer process. They explained that leadership commitment from the start of the intervention would make the intervention more successful and quicker.

*Mechanism two: establishing the intervention as an organisational priority.* District-level managers highlighted that senior managers should make the participatory intervention an organisational priority as it helps to put the employees at the centre of consideration and legitimise the importance of creating a participatory culture.

*Mechanism three:allocating necessary resources.* District-level managers highlighted that senior managers should support the intervention with an emphasis on allocating necessary resources to implement the intervention. Such resources could include financial resources, human resources, training, and infrastructural resources (e.g., communication tools).

#### 3.2.2. Contextual Factors That May Influence Leadership Commitment

Our participants highlighted three contextual factors that may influence leadership commitment.

*Contextual factor one: availability of sufficient financial resources.* District-level managers stated that the existing budget and cost constraints of the organisation working within the competitive food service industry could be a barrier for leadership commitment to cover the increased costs associated with the participatory intervention. This suggests that, to trigger leadership commitment, there should be sufficient financial resources to cover the increased costs associated with the intervention.

*Contextual factor two: low role conflict for senior managers.* District-level managers discussed that competing priorities including managing the internal affairs of the organisation and client relationships would limit senior management time to support the intervention. This implies that, to trigger leadership commitment, senior managers should have low role conflict so they can allocate time to support the intervention.

*Contextual factor three: availability of industry level resources.* District-level managers acknowledged that existing resources at the industry level would facilitate leadership commitment. At the industry level, the resources would include the Occupational Safety and Health Administration (OSHA) (which sets and enforces standards about safe and healthy working conditions, and provides training, education, outreach, and assistance) and several organisations that senior managers could consult.

#### 3.2.3. Outcomes That May Be Produced by Leadership Commitment

Our participants suggested two outcomes that may be produced by leadership commitment.

*Outcome one: improved employee engagement and commitment to their jobs.* District-level managers argued that multi-level management (including senior management) support of the intervention would improve working conditions that ultimately improve employees’ engagement and commitment to their jobs.

*Outcome two: improvedemployee health and wellbeing*. District-level managers suggested that multi-level management (including senior management) support of the intervention to improve working conditions would improve employee-perceived managerial support. This would positively correlate with improving employee health and wellbeing.

The above analyses lead to the following initial MRT.

*Initial MRT about leadership commitment: ****if*** there are sufficient financial resources in the organisation, senior managers have low role conflict, and there are industry-level resources (*contextual factors*), ***then*** leadership commitment to the intervention by being involved from the start of the intervention, establishing the intervention as an organisational priority, and allocating necessary resources (*mechanisms*), will improve employee-perceived managerial support, which will consequently improve employee health and wellbeing, job engagement, and commitment (*outcomes*).

### 3.3. Initial MRT about Communication

#### 3.3.1. Mechanisms of Communication

Our data showed that communication may operate in two ways (i.e., two mechanisms) within the food service industry intervention, as detailed below.

*Mechanism one: establishing two-way communication.* District-level managers highlighted that a mechanism should establish two-way communication (i.e., top-down and bottom-up) between managers at different levels and employees about the intervention activities.

*Mechanism two: establishing clear, precise, and specific communication about the goals, process, and content of the intervention.* Managers at different levels suggested that communication about the goals, process, and content of the intervention should be clear, precise, and specific. They suggested that the purpose of the intervention, the implementation process of the intervention, and the targeted working conditions to change by the intervention should be communicated to employees.

#### 3.3.2. Contextual Factors That May Influence Communication

Our participants highlighted five contextual factors that may influence communication.

*Contextual factor one: flexibility of organisational communication structures to accommodate both top-down and bottom-up communication flows.* Managers at different levels considered existing top-down, multi-layered communication flow in the organisation as a barrier to effective communication about the intervention. They described existing communication as a “cascade from the top down”: at the senior management level, communication was web-based and through leadership meetings; as communication cascades down, the responsibility falls on the worksite managers, often through meetings, to carry the information to employees. They acknowledged that bottom-up communication was required but missing within the organisation. This suggests that, to trigger effective communication, organisational communication structures should be flexible to accommodate both top-down and bottom-up communication flows.

*Contextual factor two: minimisedlanguage barriers.* Worksite managers reported immigrant employees from different backgrounds were employed and, therefore, language barriers in the worksites could impair effective communication regarding the intervention. This implies that, to trigger effective communication, language barriers in the organisation should be minimised.

*Contextual factor three: reasonable workloads for worksite managers.* District-level managers highlighted existing high workloads and time pressure for worksite managers as potential barriers to communication about the intervention through in-person meetings with their employees. This suggests that, to trigger effective communication, worksite managers need reasonable workloads and time allocated to have in-person communication with their employees about the intervention.

*Contextual factor four: availabilityof communication resources.* Managers at different levels reported that existing resources in the company including the safety website of the company, email, daily stand-up meetings (huddles), and formal department meetings would facilitate effective communication about the intervention.

*Contextual factor five: existinga culture of respect.* Worksite managers confirmed that the existing culture of respect in the organisation would encourage employees to communicate their ideas about improving working environment during the intervention and to provide honest feedback.

#### 3.3.3. Outcomes That May Be Produced by Communication

Our participants suggested three outcomes that may be produced by communication.

*Outcome one: improvedemployee job engagement and job satisfaction.* District-level managers suggested that communication about the content of the intervention (i.e., improving working conditions, such as by clarifying opportunities for career advancement) would improve employee job engagement and job satisfaction.

*Outcome two: improved employee health and wellbeing*. District-level managers considered communication about the intervention an important issue when implementing changes in working condition that would improve employee wellbeing and help employees maintain their health.

*Outcome three: improved employee quality of life.* District-level managers suggested that effective communication about the content of the intervention among employees and managers would improve relatedness among these different levels, create trust among them, harmonise their capabilities, and improve their daily quality of life.

The above analyses lead to the following initial MRT.

*Initial MRT about communication*: ***if*** there are flexible organisational structures for both top-down and bottom-up communication flows, few language barriers, reasonable workloads for worksite managers, necessary communication resources, a culture of respect (*contextual factors*), ***then*** establishing two-way (i.e., top-down and bottom-up) communications between managers and employees to communicate clear, precise, and specific information about the goals, process, and content of the intervention (*mechanisms*) will improve employees’ job engagement, job satisfaction, health and wellbeing, and quality of life (*outcomes*).

### 3.4. Initial MRT about Tailoring the Intervention to Fit the Organisational Context

#### 3.4.1. Mechanisms of Tailoring the Intervention to Fit the Organisational Context

Our data showed that tailoring the intervention to fit the organisational context may operate in three ways (i.e., through three mechanisms) within the food service industry intervention as specified below.

*Mechanism one:tailoring the intervention tofit individuals*. Managers at different levels acknowledged the diversity of employees in terms of their needs, attitudes, skills, competencies, and dedications and suggested that a mechanism should be tailoring the intervention to fit individuals.

*Mechanism two:tailoring the intervention to fit existingpolicies and procedures*. District-level managers proposed tailoring the intervention activities to fit existing policies and procedures. They concluded that, due to the existing top-down organisational structure, changing working conditions should be through policies and procedures verified by the senior management.

*Mechanism three:tailoring theintervention to fit existing working conditions*. Our data suggested that intervention activities should target three groups of working conditions that were perceived to be influential on employees’ safety, health, and wellbeing. These working conditions included safety practices and ergonomics (e.g., heavy lifting and carrying, injuries from cuts, burns, trips, slips, and falls), work intensity (e.g., workloads of worksite managers and employees, various shifts and schedules in worksites), and job enrichment and career advancement (e.g., role clarity and job tasks expectations, pathways for career advancement, teamwork).

#### 3.4.2. Contextual Factors That May Influence Tailoring the Intervention to Fit the Organisational Context

Our analysed data highlighted two contextual factors that may influence tailoring the intervention to fit the organisational context.

*Contextual factor one: existing good practices.* Managers at different levels highlighted that to tailor the intervention to fit the organisational context, existing good practices could be used to build intervention activities. These existing good practices would include worksite managers′ monthly safety inspections, daily and monthly safety meetings, safety committees, safety trainings, and using bulletin boards in worksites for communicating information on working conditions and safety policies and procedures.

*Contextual factor two: availability of resources.* Managers at different levels acknowledged that existing resources in the organisation could be used to tailor the intervention to fit the organisational context. These resources would include the company′s website, wellness/safety/mindfulness programmes, the Employee Assistance Programme (EAP), online resources, and training coordinators.

#### 3.4.3. Outcomes That May Be Produced by Tailoring the Intervention to Fit the Organisational Context

Our participants suggested two outcomes that may be produced by tailoring the intervention to fit the organisational context.

*Outcome one: reduced work-related injuries and musculoskeletal disorders.* District-level managers stated that a tailored intervention targeting the most relevant problematic working conditions would reduce injuries and musculoskeletal disorders associated with the job tasks completed by employees.

*Outcome two: long-term maintenance and sustainability of the intervention.* District-level managers explained that tailoring intervention activities to fit existing working conditions including physical working environment, psychosocial working environment, working culture, and relationships between the worksites and their clients would build long-term maintenance and sustainability of the intervention activities within the organisation.

The above analyses lead to the following initial MRT.

*Initial MRT about tailoring the intervention to fit the organisational context*: ***if*** there are existing good practices and necessary resources in the organisation (*contextual factors*), ***then*** a tailored intervention that fits individual employees, existing policies and procedures, and existing working conditions (*mechanisms*) will reduce employees’ work-related injuries and musculoskeletal disorders and will result in long-term maintenance and sustainability of the intervention (*outcomes*).

## 4. Discussion

In the present study, we developed an initial template containing four key process mechanisms of organisational interventions, based on the existing literature on mechanisms in organisational interventions. Then, we refined this template by developing four final MRT templates, based on qualitative empirical data from the development phase of an organisational intervention in the US food service industry. Each final template represents an initial MRT (i.e., CMO configuration), as required by realist evaluation.

The organisational intervention literature mainly has focused on causal relationships between Mechanisms–Contexts or Mechanisms–Outcomes rather than linking these together to form Contexts–Mechanisms–Outcomes relationships [6]. Regarding the initial MRT about participation, the literature provides the causal relationship between mechanisms and contexts as to trigger participation there should be reasonable workloads for employees and worksite managers [34], low employee turnover [35], high employee readiness for change [17], and availability of resources [13,36]. Regarding the causal relationship between mechanisms and outcomes, the literature shows that participation has resulted in improved employee awareness of their working conditions [13], increased employee feelings of being valued and satisfied [13,34], and improved employee management of their energy levels and fatigue [11].

Concerning the initial MRT about leadership commitment, the literature illustrates the causal relationship between mechanisms and contexts as, to trigger leadership commitment, there should be availability of sufficient financial resources [37] and availability of industrial-level resources [11]. We could not find causal evidence between the mechanism of leadership commitment and the contextual factor of low role conflict of senior management in the literature. Regarding the causal relationship between mechanisms and outcomes, the literature manifests that leadership commitment led to improved employee engagement and commitment to their jobs [38] and improved employee health and wellbeing [39].

Regarding the initial MRT about communication, the literature provides the causal relationship between mechanisms and contexts as, to trigger communication, there should be flexibility of organisational communication structures to accommodate both top-down and bottom-up communication flows [40], minimised language barriers [11], reasonable workloads for worksite managers [41], availability of resources [37], and a culture of respect [42]. Regarding the causal relationship between mechanisms and outcomes, the literature illustrates that communication produced outcomes of improved employees’ job engagement and job satisfaction and improved employee health and wellbeing [42]. We do not have causal evidence between the mechanism of communication and the outcome of improved employee quality of life, as of yet.

Finally, concerning the initial MRT about tailoring the intervention to fit the organisational context, the literature suggests that the causal relationship between mechanisms and contexts as, to trigger tailoring the intervention to fit the organisational context, there should be existing good practices in the organisation [43] and availability of resources [12]. Regarding the causal relationship between mechanisms and outcomes, the literature shows that tailoring the intervention to fit the organisational context produced outcomes of reduced employee work-related injuries and musculoskeletal disorders [44] and long-term maintenance and sustainability of the intervention [12,26].

In summary, the previous intervention studies mainly have focused on causal relationships between mechanisms and contexts or mechanisms and outcomes, the novelty of our study is providing a list of causally related context–mechanism–outcome elements in each initial MRTs that can be tested in future intervention studies.

### 4.1. Implications for Future Research and Practice

Our study has both theoretical and practical implications for planning and evaluating participatory organisational interventions. From the theory perspective, we followed realist evaluation as our theoretical approach and developed four initial MRTs. These initial MRTs provide a theoretically informed basis to focus data collection, analysis, and synthesis in future organisational intervention studies [7]. While the empirical evidence in the organisational intervention literature, to a large extent, supports the elements of our initial MRTs, these initial MRTs have not been developed or tested in a single intervention study, as required by realist evaluation [9].

From a practice point of view, our initial MRTs can be used by occupational health practitioners and organisational managers to plan and evaluate organisational interventions to improve employee health and wellbeing. In particular, our initial MRTs can be tested in organisational interventions that target immigrant employees, low-wage employees, and/or employees with low levels of autonomy; since such employees encounter relatively similar contextual factors as employees in our study, our suggested mechanisms would likely produce the above mentioned intended outcomes [11]. Our initial MRTs provide insights to occupational health practitioners and organisational managers about (1) how the mechanisms of participation, leadership commitment, communication, and tailoring the intervention to fit the organisational context can be triggered; (2) what contextual factors may influence (i.e., facilitate or impair) the operation of such mechanisms, so facilitators can be fostered, and barriers can be removed or weakened; (3) what outcomes the mechanisms may produce.

We recommend future organisational intervention studies to empirically test our initial MRTs. Testing these MRTs in an organisational intervention study helps to explore how the dynamic interactions between certain contextual factors and certain mechanisms produce certain outcomes, this helps to understand what works for whom in which circumstances in organisational interventions [7]. To test our MRTs, researchers, occupational health practitioners, and organisational managers can consult previous organisational intervention studies that followed realist evaluation [10,11,13], these studies have provided insights on how to empirically test CMO configurations using quantitative methods [13] or mixed methods (i.e., both quantitative and qualitative methods) [10,11].

### 4.2. Strengths and Limitations

This study has three strengths. First, we used two types of triangulation, namely ‘method triangulation’ by using interviews, focus groups, and observations and ‘data source triangulation’ by targeting multi-level managers and employees and using research team observations [45]. These two types of triangulation, where each method relates to a specific stakeholder, correspond to the call to explain which methods, and how these methods, were used to collect realistic data from different stakeholders [9]. Second, to develop the initial template, we focused on four critical process mechanisms that may influence the success or failure of organisational interventions; namely, participation, leadership commitment, communication, and tailoring the intervention to fit the organisational context. This focus on specific mechanisms is aligned with ‘theory adjudication’ as realist evaluation requires focusing on the most relevant mechanisms in each intervention study [9,15]. Third, we analysed and synthesised data based on template analysis incorporating the logic of retroduction. In operationalising the retroductive inferencing logic, we focused on causal relationships among contexts, mechanisms, and outcomes as made explicit by the organisational stakeholders. This approach seems fit to realist evaluation and contributes to answering the question of ‘how to construct realistic data?’ [9].

This study is not without its limitations. First, the process of analysing and synthesising realist data is based on the interpretation and judgment that a researcher/occupational health practitioner applies to data. This subjectivity is critical as the overlaps between mechanisms, contextual factors, and outcomes may create issues in explaining the causal relationship among them. In this study, we used more than one researcher (i.e., the first, second, and third author) in the process of analysing and synthesising data to minimise this limitation. Second, interviews and focus groups guides were not developed based on realist evaluation. Therefore, questions did not focus on how the intervention mechanisms could be operationalised, what contextual factors may impair or facilitate the activation of each mechanism, and what outcomes each mechanism may produce. This made developing initial MRTs challenging. Third, the triangulation of evidence was at the MRT level; we could not find different evidence from different stakeholders for each single contextual factor, mechanism, and outcome of the initial MRTs. Further, the majority of evidence is from district-level managers, implying that top managers had a better overview, compared to worksite managers and employees, about the dynamics of the prospective intervention. In realist evaluation, however, each piece of evidence that contributes to the understanding of ‘what works for whom in which circumstances’ is valued in the synthesis process [9].

## 5. Conclusions

As the first study in the organisational intervention literature to develop initial MRTs—the first phase of realist evaluation—we proposed four initial MRTs based on qualitative empirical evidence from the development phase of an organisational intervention in a large multi-national organisation in the US food service industry. The initial MRTs show how the key process mechanisms of participation, leadership commitment, communication, and tailoring the intervention to fit the organisational context can be operated, what contextual factors may influence the operation of such mechanisms, and what outcomes they may produce. As such, by formulating ‘what may work for whom in which circumstances?’, these initial MRTs provide insights into how to evaluate future interventions [7]. The initial MRTs can be tested (i.e., confirmed, refuted, or modified) using empirical data in future organisation interventions, particularly organisational interventions in organisations with immigrant employees, low-wage employees, and/or employees with low levels of autonomy.

## Data Availability

The data supporting the results presented in this study are available on reasonable request from the corresponding author. The data are not publicly available.

## References

[1] EU-OSHA Second European Survey of Enterprises on New and Emerging Risks (ESENER-2). Overview Report: Managing Safety and Health at Work. https://op.europa.eu/s/n7Wt.

[2] ILO (2001). Guidelines on Occupational Safety and Health Management Systems (ILO-OSH 2001).

[3] Nielsen K. (2013). Review Article: How can we make organizational interventions work? Employees and line managers as actively crafting interventions. Hum. Relat..

[4] Montano D., Hoven H., Siegrist J. (2014). Effects of organisational-level interventions at work on employees’ health: A systematic review. BMC Public Health.

[5] Semmer N.K. (2006). Job stress interventions and the organization of work. Scand. J. Work. Environ. Health.

[6] Roodbari H., Axtell C., Nielsen K., Sorensen G. (2021). Organisational Interventions to Improve Employees’ Health and Wellbeing: A Realist Synthesis. Appl. Psychol. Int. Rev..

[7] Nielsen K., Miraglia M. (2016). What works for whom in which circumstances? On the need to move beyond the ‘what works?’ question in organizational intervention research. Hum. Relat..

[8] Pawson R., Tilley N. (2004). Realist Evaluation. https://www.communitymatters.com.au/RE_chapter.pdf.

[9] Pawson R., Tilley N. (1997). Realistic Evaluation.

[10] Abildgaard J.S., Nielsen K., Wåhlin-Jacobsen C.D., Maltesen T., Christensen K.B., Holtermann A. (2020). ‘Same, but different’: A mixed-methods realist evaluation of a cluster-randomized controlled participatory organizational intervention. Hum. Relat..

[11] Busch C., Koch T., Clasen J., Winkler E., Vowinkel J. (2017). Evaluation of an organizational health intervention for low-skilled workers and immigrants. Hum. Relat..

[12] Nielsen K., Abildgaard J.S., Daniels K. (2014). Putting context into organizational intervention design: Using tailored questionnaires to measure initiatives for worker well-being. Hum. Relat..

[13] von Thiele S.U., Nielsen K.M., Stenfors-Hayes T., Hasson H. (2017). Using kaizen to improve employee well-being: Results from two organizational intervention studies. Hum. Relat..

[14] Roodbari H., Nielsen K., Axtell C. (2021). An integrated realist evaluation model to evaluate organisational interventions. Acad. Manag. Proc..

[15] Pawson R., Greenhalgh T., Harvey G., Walshe K. (2004). Realist Synthesis: An Introduction.

[16] Nielsen K., Noblet A., Nielsen K., Noblet A. (2018). Organizational interventions: Where are we, where do we go from here?. Organizational Interventions for Health and Well-Being: A Handbook for Evidence-Based Practice.

[17] Schelvis R.M.C., Wiezer N.M., Blatter B.M., van Genabeek J.A.G.M., Oude Hengel K.M., Bohlmeijer E.T., van der Beek A.J. (2016). Evaluating the implementation process of a participatory organizational level occupational health intervention in schools. BMC Public Health.

[18] Peters S.E., Nielsen K.M., Nagler E.M., Revette A.C., Madden J., Sorensen G. (2020). Ensuring Organization-Intervention Fit for a Participatory Organizational Intervention to Improve Food Service Workers’ Health and Wellbeing. J. Occup. Environ. Med..

[19] Sorensen G., Peters S., Nielsen K., Nagler E., Karapanos M., Wallace L., Burke L., Dennerlein J.T., Wagner G.R. (2019). Improving Working Conditions to Promote Worker Safety, Health, and Wellbeing for Low-Wage Workers: The Workplace Organizational Health Study. Int. J. Environ. Res. Public Health.

[20] Nielsen K., Randall R., Karanika-Murray M., Biron C. (2015). Assessing and Addressing the Fit of Planned Interventions to the Organizational Context. Derailed Organizational Interventions for Stress and Well-Being: Confessions of Failure and Solutions for Success.

[21] Nielsen K., Randall R. (2013). Opening the black box: Presenting a model for evaluating organizational-level interventions. Eur. J. Work Organ. Psychol..

[22] Wong G., Greenhalgh T., Westhorp G., Buckingham J., Pawson R. (2013). RAMESES publication standards: Realist syntheses. BMC Med..

[23] Wong G., Westhorp G., Manzano A., Greenhalgh J., Jagosh J., Greenhalgh T. (2016). RAMESES II reporting standards for realist evaluations. BMC Med..

[24] Tsutsumi A., Nagami M., Yoshikawa T., Kogi K., Kawakami N. (2009). Participatory Intervention for Workplace Improvements on Mental Health and Job Performance Among Blue-Collar Workers: A Cluster Randomized Controlled Trial. J. Occup. Environ. Med..

[25] Tessmer M. (2013). Planning and Conducting Formative Evaluations.

[26] Sorensen G., Peters S., Nielsen K., Stelson E., Wallace L., Burke L., Nagler E., Roodbari H., Karapanos M., Wagner G.W. (2021). Implementation of an organizational intervention to improve low-wage workers’ safety, health and wellbeing: Findings from the Workplace Organizational Health Study. BMC Public Health.

[27] Schutt R. (2018). Investigating the Social World: The Process and Practice of Research.

[28] King N., Symon G., Cassell C. (1998). Template Analysis. Qualitative Methods and Analysis in Organizational Research: A Practical Guide.

[29] Crabtree B.F., Miller W.L., Crabtree B.F., Miller W.L. (1992). A template approach to text analysis: Developing and using codebooks. Doing Qualitative Research.

[30] Glaser B., Strauss A. (1968). The Discovery of Grounded Theory: Strategies for Qualitative Research.

[31] Randall R., Cox T., Griffiths A. (2007). Participants’ accounts of a stress management intervention. Hum. Relat..

[32] Greenhalgh T., Pawson R., Wong G., Westhorp G., Greenhalgh J., Manzano A., Jagosh J. Retroduction in realist evaluation The RAMESES II Project. www.ramesesproject.org.

[33] Lincoln Y.S., Guba E.G. (1985). Naturalistic Inquiry.

[34] Nielsen K., Randall R. (2012). The importance of employee participation and perceptions of changes in procedures in a teamworking intervention. Work Stress.

[35] Arapovic-Johansson B., Wåhlin C., Hagberg J., Kwak L., Björklund C., Jensen I. (2018). Participatory work place intervention for stress prevention in primary health care. A randomized controlled trial. Eur. J. Work Organ. Psychol..

[36] Abildgaard J.S., Nielsen K., Sverke M. (2018). Can job insecurity be managed? Evaluating an organizational-level intervention addressing the negative effects of restructuring. Work Stress.

[37] Schneider A., Wehler M., Weigl M. (2019). Effects of work conditions on provider mental well-being and quality of care: A mixed-methods intervention study in the emergency department. BMC Emerg. Med..

[38] Nielsen K., Antino M., Rodríguez-Muñoz A., Sanz-Vergel A. (2021). Is it me or us? The impact of individual and collective participation on work engagement and burnout in a cluster-randomized organisational intervention. Work Stress.

[39] Niks I., de Jonge J., Gevers J., Houtman I. (2018). Work Stress Interventions in Hospital Care: Effectiveness of the DISCovery Method. Int. J. Environ. Res. Public Health.

[40] Dollard M.F., Gordon J.A. (2014). Evaluation of a participatory risk management work stress intervention. Int. J. Stress Manag..

[41] Nielsen K., Randall R. (2009). Managers’ Active Support when Implementing Teams: The Impact on Employee Well-Being. Appl. Psychol. Health Well-Being.

[42] DeJoy D.M., Wilson M.G., Vandenberg R.J., McGrath-Higgins A.L., Griffin-Blake C.S. (2010). Assessing the impact of healthy work organization intervention. J. Occup. Organ. Psychol..

[43] Yoshikawa T., Ogami A., Muto T. (2013). Evaluation of participatory training in managing mental health for supervisory employees in the financial industry. J. Hum. Ergol..

[44] Haslam C., Kazi A., Duncan M., Clemes S., Twumasi R. (2019). Walking Works Wonders: A tailored workplace intervention evaluated over 24 months. Ergonomics.

[45] Patton M.Q. (1999). Enhancing the quality and credibility of qualitative analysis. Health Serv. Res..

